# Selective pressure of antibiotics on ARGs and bacterial communities in manure-polluted freshwater-sediment microcosms

**DOI:** 10.3389/fmicb.2015.00194

**Published:** 2015-03-11

**Authors:** Wenguang Xiong, Yongxue Sun, Xueyao Ding, Mianzhi Wang, Zhenling Zeng

**Affiliations:** National Laboratory of Safety Evaluation (Environmental Assessment) of Veterinary Drugs, College of Veterinary Medicine, South China Agricultural UniversityGuangzhou, China

**Keywords:** antibiotics, selective pressure, antibiotic resistance genes, bacterial community, microcosms

## Abstract

The aim of this study was to investigate selective pressure of antibiotics on antibiotic resistance genes (ARGs) and bacterial communities in manure-polluted aquatic environment. Three treatment groups were set up in freshwater-sediment microcosms: tetracyclines group, sulfonamides group and fluoroquinolones group. Sediment and water samples were collected on day 14 after treatment. Antibiotic concentrations, ARGs abundances and bacterial community composition were analyzed. Antibiotic concentrations were determined by ultra-performance liquid chromatography-electrospray tandem mass spectrometry. ARGs abundances were quantified by real time quantitative PCR. Bacterial community composition was analyzed based on amplicon sequencing. Of the three classes of antibiotics analyzed in the treatment groups, accumulation amounts were tetracyclines> fluoroquinolone> sulfonamides in the sediment samples, while they were sulfonamides> fluoroquinolone> tetracyclines in the water samples. In the treatment groups, the relative abundances of some tet resistance genes [*tet*(W) and *tet*(X)] and plasmid-mediated quinolone resistance (PMQR) genes [*oqx*(B) and *aac(6′)-Ib*] in sediment samples were significantly higher than those in the paired water samples. Tetracyclines significantly selected the bacterial classes including *Gammaproteobacteria*, *Clostridia*, and the genera including *Salmonella*, *Escherichia/Shigella*, *Clostridium*, *Stenotrophomonas* in sediment samples. The significant selection on bacterial communities posed by sulfonamides and fluoroquinolones was also observed. The results indicated that sediment may supply an ideal setting for maintenance and persistence of tet resistance genes [*tet*(W) and *tet*(X)] and PMQR genes [*oqx*(B) and *aac(6′)-Ib*] under antibiotic pollution. The results also highlighted that antibiotics significantly selected specific bacterial communities including the taxa associated with opportunistic pathogens.

## Introduction

Antibiotic resistance genes (ARGs) in the environment are of great concern, since they can be acquired by human commensal bacteria and clinical pathogens. Persistence and spread of environmental ARGs can be promoted by animal production activities (Li et al., 2012; Zhu et al., 2013). Large amounts of various antibiotics have been widely used for disease prevention, disease treatment and growth promotion in animal feedlots. The production of antibiotics used in animal feedlots was approximately 9200 tons in the USA in 2003 (Arikan et al., 2007), and about 6000 tons of veterinary antibiotics was used annually in China (Zhao et al., 2010). High concentrations of antibiotics were observed in the manure from treated swine (e.g., total concentrations of tetracyclines and sulfonamides were 30.97 and 18.59 mg kg^−1^, respectively) (Ji et al., 2012) and swine wastewater (e.g., oxytetracycline and tylosin reached up to 2.02 and 2.10 mg L^−1^, respectively) (Angenent et al., 2008; Ben et al., 2013). Antibiotics discharged from animal feedlots promote the emergence and spread of environmental ARGs (Li et al., 2012).

Antibiotics pose the primarily selective pressure on ARGs. Evidence suggested that antibiotics in both high and low concentrations (below the minimal inhibitory concentrations) promote the emergence and persistence of antibiotic resistance (Gullberg et al., 2011). Furthermore, antibiotics facilitate the horizontal gene transfer of ARGs among different bacteria (Hong et al., 2013a). The bacteria acquiring ARGs may be enriched under antibiotic selection, leading to the change of bacterial communities. Antibiotics change bacterial communities by decreasing susceptible bacterial groups while increasing resistant bacterial ones (Cantón and Ruiz-Garbajosa, 2011).

The wastewater discharged from animal feedlots can reach downstream, and contaminates groundwater supply. ARGs and resistant bacteria in natural water system could spread into drinking water, and posed health risk via food chain (Walsh et al., 2011; Jiang et al., 2013). ARGs and fecal-origin bacteria (including resistant bacteria and opportunistic pathogens) have been observed in manure-polluted aquatic system (Barton, 2014; Hsu et al., 2014). Previous studies have investigated ARGs abundance and bacterial community composition in manure-polluted aquatic systems (Hong et al., 2013b; Brooks et al., 2014; Lu and Lu, 2014). As we all know, however, the environments in field studies are very complex. Besides antibiotics, several other factors such as rainfall, sunlight, and seasonal variations all influence ARGs abundance and/or bacterial communities (Engemann et al., 2008; Novo et al., 2013). Therefore, the need for complementary studies is underscored to provide direct evidence on selective pressure of antibiotics on ARGs and bacterial communities in microcosms by suppressing the factors mentioned above.

The aim of this study was to investigate selective pressure of antibiotics on ARGs and bacterial communities in a manure-polluted aquatic environment. Quantitative data with direct evidences on selective pressure of antibiotics on ARGs and bacterial communities in freshwater-sediment microcosms were provided for the first time, by (1) detecting nine antibiotics including tetracyclines, sulfonamides and fluoroquinolones (2) quantifying various ARGs including tet resistance genes, sul resistance genes and plasmid-mediated quinolone resistance (PMQR) genes (3) analyzing bacterial community composition.

## Materials and methods

### Treatments and microcosms setup

Sediment and water were collected from the Liuxi River in Guangzhou, China. The sediment had a moisture content of 48.7%, with a silt loam (73% silt, 15% sand, and 12% clay). The water had pH of 6.5 and dissolved oxygen of 8.7 mg L^−1^. Fresh manure was collected from a representative swine feedlot with a population of more than 1000 swine. The manure had pH of 7.35 and dry matter of 15.3%. Weighted 100 g of sediment and 800 mL of water were added in each beaker with the capacity of 1000 mL (shown in Supplemental Material sections Figure S1). Weighted 1 g of manure was added in each beaker with or without antibiotics, which served as treatment and control groups, respectively. Three treatment groups in three replications were set up as follows: tetracyclines group (chlorotetracycline, oxytetracycline, and doxycycline), sulfonamides group (sulfamethoxydiazine, sulfamethazine, and sulfamethoxazole) and fluoroquinolones group (enrofloxacin, ciprofloxacin, and norfloxacin). The concentration of individual antibiotics was 1 mg L^−1^. All beakers were incubated in the dark at 20°C. The river water was added to the beakers twice a week to compensate for the weight loss of the microcosms. Sediment and water samples were collected on day 14 after treatment.

### UPLC-MS/MS analysis

Water samples were filtered through 0.45 μm glass fiber filters to remove suspended solids. Weighted 1 g of sediment and 50 mL of water were freeze-dried at −80°C, respectively. The freeze-dried sediment and water samples were spiked with 5 mL of extraction buffer including acetonitrile/phosphate (v/v = 1:1 and pH = 3.0). Solid phase extraction was conducted according to the method of Luo et al. (2010). Antibiotic concentrations were determined by ultra-performance liquid chromatography-electrospray tandem mass spectrometry (UPLC-MS/MS). Concentrated extracts were separated on an Agilent 1200 liquid chromatograph (Santa Clara, CA, USA) by using a Waters Quattro Micro triple quadrupole mass spectrometer in multiple reactions monitoring mode with electrospray ionization in positive-ion mode (CityMilford, MA, USA).

### PCR and qPCR analysis

Sediment and water DNA were extracted by using Power Soil and Water DNA Kit (Mo Bio Laboratories, Inc., CA, USA) according to the manufacturer's instructions, respectively. The presences of various ARGs including tet resistance genes [*tet*(M), *tet*(O), *tet*(W), *tet*(S), *tet*(Q), *tet*(X), and *tet*(B/P)], sul resistance genes [*sul*(1), *sul*(2), and *sul*(3)], PMQR genes [*oqx*(A), *oqx*(B), *aac*(6′)*-Ib*, *qnr*(S), and *qep*(A)] were determined by PCR. PCR was performed by using TaKaRa Ex Taq PCR Kit (TaKaRa, Dalian, China) according to the manufacturer's instructions. PCR products were analyzed by gel electrophoresis using 1% (w/v) agarose in 1 × TAE buffer. Positive products of PCR were cloned, extracted and sequenced for standard curves of real time quantitative PCR (qPCR). Extracted DNA was diluted by 1/100 fold to minimize the inhibition of sample matrix. qPCR was performed by using SYBR Premix Ex Taq II (TaKaRa, Dalian, China) according to the manufacturer's instructions. The negative and positive controls were both conducted in each run. The specificity was verified by melting curves and gel electrophoresis. The efficiency of each gene (91–102%) was checked with R^2^ values more than 99.2% for all calibration curves. Primer sequences, amplicon size and annealing temperature are described in Supplementary Material sections Table S1. Given the temporal variations caused by total bacterial community and overall extraction efficiencies, the copies of ARGs were normalized to the 16S rRNA gene copies (ARGs copies/16S rRNA gene copies, defined as relative abundance).

### Amplicon sequencing

The DNA extracted from sediment and water samples was further analyzed for bacterial community composition. Universal bacterial primers (F: 5′- ACTCAAATGAATTGACGGGG- 3′) and (R: 5′- GCTCGTTGCGGGACTTAA- 3′) were used to amplify V6 region in 16S rRNA gene (Baker et al., 2003). The details regarding the procedure of amplicon sequencing were described previously (Xiong et al., 2015). DNA was extracted from three replications for each sample. The PCR products from the three replications were pooled together in equimolar ratios for amplicon sequencing. Amplicon sequencing was performed on Ion Torrent Personal Genome Machine with Ion 316 chip. After sequencing, RDP Classifier tool was used to determine the phylogenetic classification of sequences (Cole et al., 2014). Raw sequences were submitted to Sequence Read Archive database in NCBI (accession No. **PRJNA269563**).

### Statistical analyses

Significant differences of relative abundance of ARGs at *p* < 0.05 between sediment and paired water were analyzed by ANOVA/LSD post hoc test using SPSS 18.0. Differential abundances of bacterial taxa between the treatment and control groups were compared by using Fisher's exact test at the statistical difference of *p* < 0.05.

## Results

### Antibiotic concentrations

**Figure 1:**
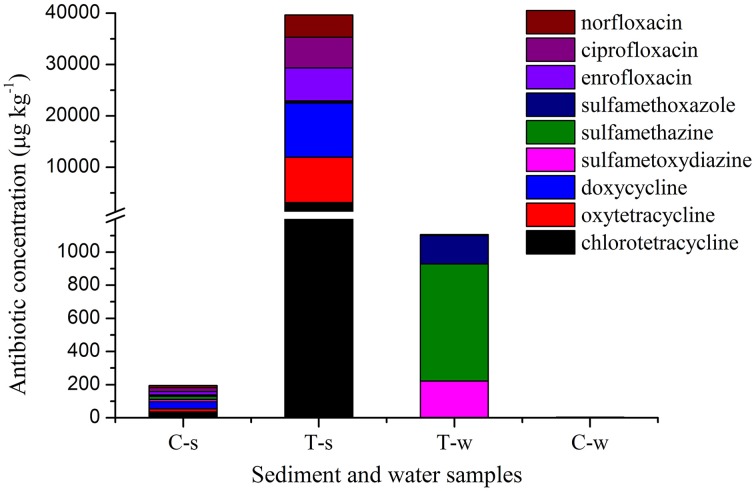
**Antibiotic concentrations in sediment and water samples in different groups**. C-s: sediment in the control group; T-s: sediment in the treatment groups including tetracyclines, sulfonamides and fluoroquinolones groups, respectively. T-w: water in the treatment groups including tetracyclines, sulfonamides and fluoroquinolones groups, respectively. C-w: water in the control group. The break in Y axis was from 1200 to 1300 μg kg^−1^.

Antibiotic concentrations are shown in Figure 1 and Supplemental Material sections Table S2. In the treatment groups, the concentrations of individual tetracycline and fluoroquinolone in sediment samples were 10333–30344 and 2909–3308 fold of those in the paired water samples, while the concentrations of individual sulfonamide in sediment samples were 0.23–0.82 fold of those in the paired water sample. Of sediment samples analyzed in this study, the concentrations of individual tetracycline and fluoroquinolone in the treatment groups were 89–440 and 250–331 fold of those in the control group, while the concentrations of individual sulfonamide in the treatment group were 8–17 fold of those in the control group. Of water samples analyzed in this study, the concentrations of individual tetracycline and fluoroquinolone in the treatment groups were 0.6–8.1 and 5.9–38 fold of those in the control group, while the concentrations of individual sulfonamide in the sulfonamides group were 246-2630 fold of those in the control group.

### Antibiotic resistance genes

**Figure 2:**
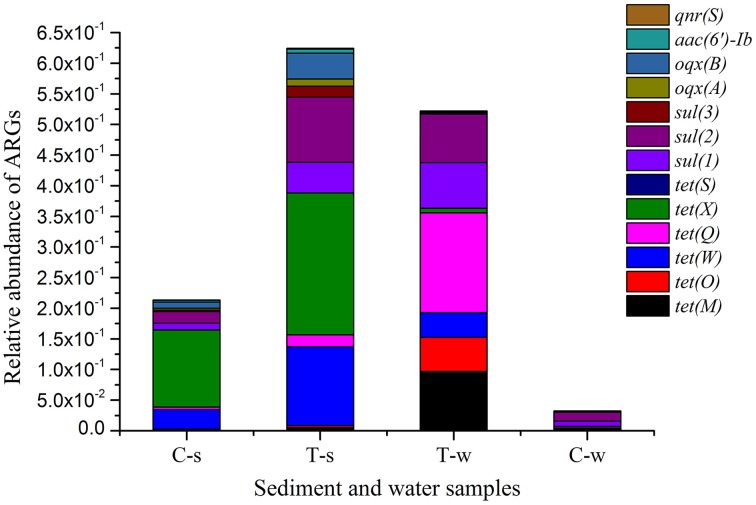
**Relative abundance of antibiotic resistance genes in sediment and water samples in different groups**. *tet*(B/P) and *qep*(A) are not shown, since they were absent in all sediment and water samples. C-s: sediment in the control group; T-s: sediment in the treatment groups including tetracyclines, sulfonamides and fluoroquinolones groups, respectively. T-w: water in the treatment groups including tetracyclines, sulfonamides and fluoroquinolones groups, respectively. C-w: water in the control group.

Relative abundances of ARGs are shown in Figure 2 and Supplemental Material sections Table S3. Of sediment samples analyzed in this study, the relative abundances of tet resistance genes [*tet*(M), *tet*(O), *tet*(W), *tet*(Q), *tet*(X), and *tet*(S)], sul resistance genes [*sul*(1), *sul*(2), and *sul*(3)] and PMQR genes [*oqx*(A), *oqx*(B), *aac(6′)-Ib*, and *qnr*(S)] in the treatment groups were 1.8–4.5, 4.5–7.2, and 1.8–4.2 fold of those in the control group, respectively. Of water samples analyzed in this study, the relative abundances of tet resistance genes [*tet*(M), *tet*(O), *tet*(W), *tet*(Q), and *tet*(X)], sul resistance genes [*sul*(1), *sul*(2), and *sul*(3)] and PMQR genes [*oqx*(A), *oqx*(B), and *aac(6′)-Ib*] in the treatment groups were 15.7–67.5, 0.8–9.0, and 1.7–7.7 fold of those in the control group, respectively. Some ARGs were absent in the control group, and they were also absent in the treatment groups, such as *tet*(S), *qnr*(S) in water samples, and *tet*(B/P), *qep*(A) in sediment and water samples. In the treatment groups, the relative abundances of some tet resistance genes [*tet*(W) and *tet*(X)] and PMQR genes [*oqx*(B) and *aac(6′)-Ib*] in sediment samples were significantly higher than those in the paired water samples, while the relative abundances of other tet resistance genes [*tet*(M), *tet*(O), and *tet*(Q)] in sediment samples were significantly lower than those in the paired water samples. There were no statistical differences of relative abundances of all sul resistance genes [*sul*(1), *sul*(2), and *sul*(3)] between the sediment and paired water samples in the sulfonamides group.

### Bacterial community composition

**Figure 3:**
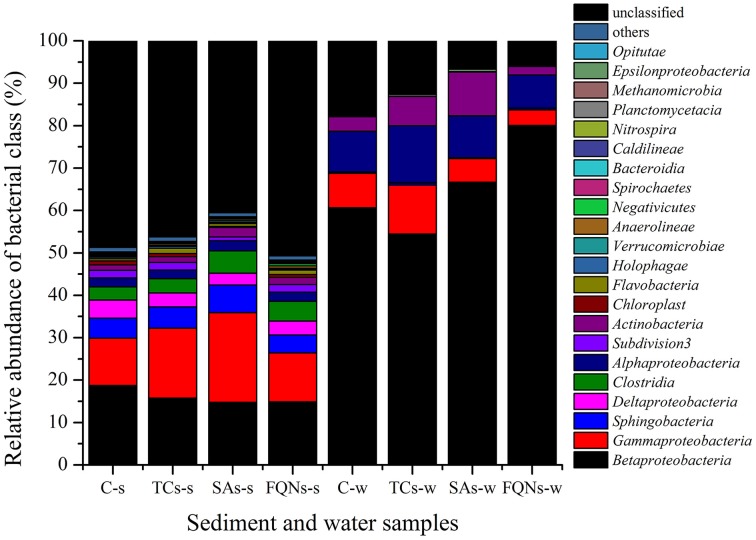
**Relative abundances of bacterial classes in sediment and water samples in different groups**. C-s: sediment in the control group; TCs-s: sediment in the tetracyclines group; SAs-s: sediment in the sulfonamides group; FQNs-s: sediment in the fluoroquinolones group. C-w: water in the control group; TCs-w: water in the tetracyclines group; SAs-w: water in the sulfonamides group; FQNs-w: water in the fluoroquinolones group.

A total of 234, 446 reads with 29, 305 high quality sequences per sample were obtained. As shown in Figure 3 and Table 1, the dominant classes were *Betaproteobacteria* (14.76–18.75%), *Gammaproteobacteria* (11.14–21.16%), *Sphingobacteria* (4.18–6.47%) in all sediment samples, and *Betaproteobacteria* (54.44–80.06%), *Gammaproteobacteria* (3.64–11.52%), *Alphaproteobacteria* (7.77–13.38%) in all water samples. Compared to the control group, antibiotic groups significantly selected for various bacterial classes. As shown in Table 1, for example, tetracyclines significantly selected for *Gammaproteobacteria*, *Clostridia* in the sediment, and *Gammaproteobacteria*, *Sphingobacteria*, *Deltaproteobacteria* in the paired water; sulfonamides significantly selected for *Gammaproteobacteria*, *Sphingobacteria*, *Clostridia* in the sediment, and *Betaproteobacteria*, *Actinobacteria* in the paired water. All the significant variations of relative abundances of bacterial classes are details in Table 1. Based on the sequences, a further evaluation at genus levels revealed that the antibiotics used in this study selected for some bacterial genera that might be associated with opportunistic pathogens. For example, tetracyclines significantly selected for *Salmonella*, *Escherichia/Shigella*, *Clostridium*, *Stenotrophomonas*, and sulfonamides significantly selected for *Acinetobacter*, *Escherichia/Shigella*, *Clostridium*, *Stenotrophomonas* in the treated sediment compared to the control sediment.

**Table 1:** **Significantly variation^a^ barcode of relative abundances of bacterial classes, and genera associated with opportunistic pathogens in treated samples compared to control samples**.

**Bacterial taxa**	**Samples^b^ and abundance range^c^**							
**Classes**	**TCs-s**	**SAs-s**	**FQNs-s**	**Abundance range (%)**	**TCs-w**	**SAs-w**	**FQNs-w**	**Abundance range (%)**
*Betaproteobacteria*	↓	↓	↓	14.76–18.75	↓	↑	↑	54.44–80.06
*Gammaproteobacteria*	↑	↑	–	11.14–21.16	↑	↓	↓	3.64–11.52
*Sphingobacteria*	–	↑	↓	4.18–6.47	↑	↓	–	0.18–0.49
*Deltaproteobacteria*	↓	↓	↓	2.83–4.29	↑	–	–	0.03–0.08
*Clostridia*	↑	↑	↑	3.08–5.24	–	–	–	0.02–0.04
*Alphaproteobacteria*	–	↑	–	2.01–2.44	↑	–	↓	7.77–13.38
*Subdivision3*	–	↓	–	0.84–1.82	–	–	–	0.02–0.05
*Actinobacteria*	–	↑	↑	1.3–2.26	↑	↑	↓	1.94–10.32
*Chloroplast*	–	↓	↓	0.21–0.89	/	/	/	/
*Flavobacteria*	↑	↑	↑	0.5–1.19	–	–	–	0–0.02
*Holophagae*	–	–	↓	0.19–0.46	/	/	/	/
*Verrucomicrobiae*	↓	–	↓	0.23–0.35	↑	–	↑	0.01–0.29
*Anaerolineae*	–	↑	↑	0.27–0.59	/	/	/	/
*Negativicutes*	↓	–	↑	0.17–0.46	–	↑	–	0–0.05
*Spirochaetes*	↓	↓	↑	0.01–0.25	/	/	/	/
*Bacteroidia*	↓	–	↑	0.04–0.24	–	–	–	0–0.01
*Caldilineae*	↑	–	–	0.02–0.09	/	/	/	/
*Nitrospira*	↑	↑	↑	0.05–0.13	/	/	/	/
*Planctomycetacia*	↑	↑	↑	0.01–0.1	↑	–	↑	0.04–0.46
*Methanomicrobia*	–	–	↑	0.01–0.03	/	/	/	/
*Epsilonproteobacteria*	–	↓	–	0–0.03	↑	↑	↓	0–0.57
*Opitutae*	↓	–	–	0–0.05	/	/	/	/
*Chlamydiae*	↓	–	–	0–0.02	/	/	/	/
**GENERA**								
*Stenotrophomonas*	↑	↑	↑	1.4–2.31	/	/	/	/
*Clostridium*	–	↑	↑	1.11–2.17	/	/	/	/
*Escherichia/Shigella*	↑	↑	–	0.18–0.64	↑	/	↑	0–0.09
*Acinetobacter*	–	↑	↑	0.16–0.42	↑	↑	↑	0.08–2.41
*Salmonella*	↑	–	–	0.05–0.11	/	/	/	/
*Treponema*	↓	↓	↑	0.01–0.25	/	/	/	/
*Arcobacter*	/	/	/	/	↑	↑	/	0–0.13

a *↑: significant increase. ↓: significant decrease. -: no significance. /: not detected*.

b *TCs-s: sediment in the tetracyclines group; SAs-s: sediment in the sulfonamides group; FQNs-s: sediment in the fluoroquinolones group. TCs-w: water in the tetracyclines group; SAs-w: water in the sulfonamides group; FQNs-w: water in the fluoroquinolones group*.

c *Relative abundance range of bacterial classes, and genera associated with opportunistic pathogens between treatment and control samples*.

## Discussion

Of the three classes of antibiotics analyzed in the treatment groups, accumulation amounts were tetracyclines> fluoroquinolone> sulfonamides in the sediment samples, while they were sulfonamides> fluoroquinolone> tetracyclines in the water samples. Tetracyclines and fluoroquinolones were mainly accumulated in sediment compared to water. The difference in the distribution of antibiotics should be dependent on antibiotic chemical stability and partition characteristics (Hari et al., 2005). Tetracyclines highly absorbed to organic matter (Hund-Rinke et al., 2004), and fluoroquinolones were strongly absorbed to sediment, soil and dissolved organic matter (Córdova-Kreylos and Scow, 2007; Babić et al., 2013). The concentrations of individual sulfonamide in the sediment and paired water were at the same order of magnitude in low concentrations in the sulfonamides group. Given mass balance in the microcosm, most sulfonamides might strongly bind to sediment and became nonextractable, which was also observed for sulfamethazine (Carstens et al., 2013).

Antibiotics pose primarily selective pressure on ARGs via evolutionary mutations and/or horizontal gene transfer. Selection strength posed by antibiotics is an important parameter contributing to antibiotic resistance (Oz et al., 2014). Previous studies have reported the correlation between antibiotic concentrations and ARGs abundances in water bodies (Li et al., 2012; Huerta et al., 2013). In this study, although detected concentrations of tetracyclines, sulfonamides and fluoroquinolones were significantly different in the treated sediments, these three classes of antibiotics posed the comparable selection on sediment ARGs (reflected by 1.8–4.5, 4.5–7.2, and 1.8–4.2 fold of the relative abundance of detected tet resistance genes, sul resistance genes and PMQR genes in the treated sediments compared to the control sediment, respectively). On the contrast, the selective pressure on water ARGs was variable (reflected by 15.7–67.5, 0.8–9.0, and 1.7–7.7 fold of the relative abundance of detected tet resistance genes, sul resistance genes and PMQR genes in the treated water compared to the control water, respectively). It was unexpected that the selective pressure on *sul*(3) was not observed in the sulfonamides treated water (4.6 × 10^−4^) compared to the control water (5.8 × 10^−4^) (Supplemental Material sections Table S3). Sulfonamides did not select for *sul*(3) in the water environment, which might partially explain the phenomenon that prevalence and abundance of *sul*(3) were significantly lower than those of *sul*(1) and *sul*(2) in other studies (Jiang et al., 2013; Hsu et al., 2014). Antibiotics could not significantly select the ARGs that did not exist initially. *Tet*(S), *qnr*(S) in water samples, and *tet*(B/P), *qep*(A) in sediment and water samples were absent in the control group, and they were also absent in the groups with corresponding antibiotic treatment.

Wastewater discharged into the receiving water bodies resulted in a high accumulation of ARGs in sediments adjacent to the contamination source (Czekalski et al., 2014). Compared to water, sediment may supply an ideal setting for maintenance and persistence of ARGs including tet resistance genes [*tet*(W) and *tet*(X)] and PMQR genes [*oqx*(B) and *aac(6′)-Ib*] in this study, since the relative abundances of these ARGs significantly increased in sediment samples than those in the paired water samples in the treatment groups (Figure 2 and Supplemental Material sections Table S3). First, significantly higher concentrations of tetracycline and fluoroquinolone (Figure 1) in sediment samples than those in the paired water samples may pose stronger selection on these ARGs. Second, higher diversities of bacterial communities in sediment than those in water (Figure 3) may facilitate the horizontal gene transfer of those ARGs among the sediment bacterial population. Chen et al. (2013) also found the similar result that the total abundance of tet resistance genes in sediments was at least 100 times higher than that in water. However, this case was not for other tet resistance genes [*tet*(M), *tet*(O), and *tet*(Q)] and sul resistance genes [*sul*(1), *sul*(2), and *sul*(3)] in this study. Bacterial hosts harboring *tet*(M), *tet*(O), and *tet*(Q) might be inhibited in sediment due to the pharmacological activities of tetracyclines. The comparable concentrations of sulfonamides in sediment and paired water (Figure 1) may pose comparable selective pressure on *sul*(1), *sul*(2), and *sul*(3), leading to no statistical differences of relative abundances of these sul resistance genes between the sediment and paired water samples.

A large proportion of animal waste is discharged into surrounding rivers and groundwater system via drainage ditches. Gastrointestinal bacteria including animal commensal bacteria and opportunistic pathogens are introduced into water system by animal waste, and exchange genetic information (e.g., resistance) with indigenous bacteria. Furthermore, antibiotics introduced by animal waste pose selective pressure on indigenous bacterial communities, by selecting a resistant subpopulation within a susceptible bacterial population. Obviously, significant selection of antibiotics on bacterial communities was evident at the class level (Figure 3 and Table 1). Hsu et al. (2014) also observed that riverine microbial community composition was altered by the wastewater containing antibiotics discharged from swine feedlots. The enrichment of specific bacteria could be the consequence of antibiotic treatment at the expense of other bacteria (Antonopoulos et al., 2009). Evidences on antibiotic selection on bacterial communities were also found in aquatic system with different pollution sources in previous studies. For example, The correlation between abundance of *Gammaproteobacteria*, *Clostridia*, *Bacteroidia* and antibiotic residues was found in a hospital-urban wastewater treatment plant system (Varela et al., 2014); *Deltaproteobacteria*, *Bacilli*, *Clostridia*, and *Epsilonproteobacteria* might be specifically associated with antibiotic (penicillin G and oxytetracycline, respectively) polluted rivers (Li et al., 2011); and the concentrations of antibiotics including tetracyclines, penicillins, sulfamides, and quinolones were positively correlated with the abundance of *Epsilonproteobacteria* and negatively with *Gamma*-, *Beta*- *proteobacteria* and *Firmicutes* in treated wastewater (Novo et al., 2013). Different responses of bacterial communities to antibiotic pollution were observed between the above and present studies, probably due to the different pollutants and bacterial community composition in the environment with different pollution sources.

The response of the same taxa to the same antibiotics exposure was different between the sediment and paired water samples. For example, *Betaproteobacteria* significantly enriched in the sediment while it significantly decreased in the paired water in sulfonamides group compared to the control group. The similar results were also observed for *Betaproteobacteria* in the microcosm exposed to fluoroquinolones, and *Gammaproteobacteria* in the microcosm exposed to sulfonamides. Different bacterial community composition in different physical environments (e.g., sediment vs. water) may lead to the variable responses of bacterial taxa to antibiotic pollution, which indicated that physical environments should be considered in future studies of antibiotic selection on bacterial communities. The reasons may be explained as follows: first, the bacterial communities in sediment and water may harbor different intrinsic or innate resistance. Second, sediment and water may have different physical diffusion barriers like biofilms to prevent antibiotics from reaching their targets (Paraje, 2011). The response of bacteria to antibiotics in sediment and water should be the results of an intricate mixture of intrinsic and extrinsic (antibiotic-induced) factors.

It should be noted that the antibiotics significantly selected some genera associated with opportunistic pathogens. These genera possess the probability of becoming antibiotic-resistant pathogens, which have significant implications for human health. Among these, the genus *Acinetobacter* was present in the manure-polluted microcosms, and was significantly selected by sulfonamides and fluoroquinolones in sediment. Although some species of *Acinetobacter* are environmental commensal bacteria, other species belonging to *Acinetobacter* possessed the potential to exhibit increasing virulence, carry multidrug resistance, and cause several nosocomial infections (Doughari et al., 2011). Besides *Acinetobacter*, *Clostridium* was also significantly selected by sulfonamides and fluoroquinolones in sediment. Some species, such as *Clostridium difficile* causes diarrhea and colitis with increasing incidence, severity, and mortality (Khanna et al., 2011); and *Clostridium perfringens* represents a fecal indicator that serves as a human pathogen (Mueller-Spitz et al., 2010). However, we could not determine if *Clostridium difficile* and *Clostridium perfringens* were present, since the metagenomic approach did not allow identification at the species level. *Acinetobacter* and *Clostridium* were also present in groundwater ecosystems adjacent to pig feedlots (Hong et al., 2013b). Lastly, we found that tetracyclines significantly selected *Salmonella*, of which many strains from swine have been observed to be resistance to multi-drug including tetracyclines, streptomycin, sulphonamide-trimethoprim and ampicillin (Barton, 2014; Gomes-Neves et al., 2014). Antibiotics posed selective pressure on clinically relevant bacteria, which may increase resistant prevalence (Tello et al., 2012).

This study provided quantitative data on selective pressure of antibiotics on ARGs and bacterial community composition in manure-polluted aquatic environment. The results supported the conclusions as follows: (1) Positive selection on tet resistance genes [*tet*(M), *tet*(O), *tet*(W), *tet*(Q), and *tet*(X)], sul resistance genes [*sul*(1) and *sul*(2)] and PMQR genes [*oqx*(A), *oqx*(B), and *aac(6′)-Ib*] posed by corresponding antibiotics was observed in the sediment and water, except *sul*(3) in water. (2) Antibiotics could not significantly select the ARGs that did not exist initially. (3) tet resistance genes [*tet*(W) and *tet*(X)] and PMQR genes [*oqx*(B) and *aac(6′)-Ib*] significantly enriched in sediment rather than water under antibiotic pollution. (4) Antibiotics significantly selected for specific bacterial community including the taxa associated with opportunistic pathogens. In total, the significant antibiotic selection on most ARGs suggested that ARGs accumulation in the manure-polluted freshwater-sediment environments (e.g., downstream rivers adjacent to animal feedlots) can be reduced by minimizing antibiotic input by manure application. Also the significant enrichment of bacterial communities posed by antibiotics in this study should be noted, since these selected bacterial communities (particularly the taxa associated with opportunistic pathogens) potentially served as reservoirs of ARGs in realistic manure-polluted environment. Although this study focused on selected ARGs and bacterial communities, respectively, further studies should focus on the specific ARGs linked to certain bacterial groups by using metagenomic anaysis.

### Conflict of interest statement

The authors declare that the research was conducted in the absence of any commercial or financial relationships that could be construed as a potential conflict of interest.
