# Supplementary material for: Selective pressure of antibiotics on ARGs and bacterial communities in manure-polluted freshwater-sediment microcosms
Source: Front Microbiol. 2015 Mar 11;6:194. doi: 10.3389/fmicb.2015.00194 (PMC4356103; doi:10.3389/fmicb.2015.00194)
Supplement: Supplementary file 1 [file Presentation1.PDF]

Supplemental materials: Selective pressure of antibiotics on ARGs and bacterial communities in manure-polluted freshwater-sediment microcosms

Wenguang Xiong†, Yongxue Sun†, Xueyao Ding, Mianzhi Wang, and Zhenling Zeng*

National Laboratory of Safety Evaluation (Environmental Assessment) of Veterinary Drugs, College of Veterinary Medicine, South China Agricultural University, Guangzhou, China

* **Corresponding:**
Zhenling Zeng, National Laboratory of Safety Evaluation (Environmental Assessment) of Veterinary Drugs, College of Veterinary Medicine, South China Agricultural University, 483 Wushan Road, Guangzhou 510642, China. E-mail: zlzeng@scau.edu.cn

† Wenguang Xiong and Yongxue Sun contributed equally to this paper.

Table S1 Primers, amplicon size and annealing temperature for PCR and qPCR in the study.

Target genes	Primers for PCR and qPCR	Amplicon size (bp)	Annealing temperature (□) for PCR and qPCR	Reference
tet(M)	ACAGAAAGCTTATTATATAAC<br>TGGCGTGTCTATGATGTTCAC	171	49/55	(Aminov et al., 2001)
tet(O)	ACGGARAGTTTATTGTATACC<br>TGGCGTATCTATAATGTTGAC	171	53.7/55	(Aminov et al., 2001)
tet(W)	GAGAGCCTGCTATATGCCAGC<br>GGGCGTATCCACAATGTTAAC	168	61/55	(Aminov et al., 2001)
tet(S)	GAAAGCTTACTATACAGTAGC<br>AGGAGTATCTACAATATTTAC	169	45.6/55	(Aminov et al., 2001)
tet(Q)	AGAATCTGCTGTTTGCCAGTG<br>CGGAGTGTCAATGATATTGCA	169	56.3/55	(Aminov et al., 2001)
tet(X)	CAATAATTGGTGGTGGACCC<br>TTCTTACCTTGGACATCCCG	468	53.7/60	(Ng et al., 2001)
tet(B/P)	AAAACTTATTATATTATAGTG<br>TGGAGTATCAATAATATTCAC	169	46.9/55	(Aminov et al., 2001)
sul(1)	CGCACCGGAAACATCGCTGCAC<br>TGAAGTTCCGCCGCAAGGCTCG	163	56.3/55	(Pei et al., 2006)
sul(2)	TCCGGTGGAGGCCGGTATCTGG<br>CGGGAATGCCATCTGCCTTGAG	191	58.6/55	(Pei et al., 2006)
sul(3)	TCCGTTCAGCGAATTGGTGCAG<br>TTCGTTCACGCCTTACACCAGC	128	61/55	(Pei et al., 2006)
qep(A)	CCAGCTCGGCAACTTGATAC<br>ATGCTCGCCTTCCAGAAAA	570	58.6/55	(Xia et al., 2010)
oqx(A)	CTCGGCGCGATGATGCT<br>CCACTCTTCACGGGAGACGA	392	61/55	(Kim et al., 2009)
oqx(B)	TCCTGATCTCCATTAACGCCCA<br>ACCGGAACCCATCTCGATGC	131	61/55	(Kim et al., 2009)
aac(6')-Ib	TTGCGATGCTCTATGAGTGGCTA<br>CTCGAATGCCTGGCGTGTTT	482	56.3/55	(Park et al., 2006)
qnr(S)	GCAAGTTCATTGAACAGGGT<br>TCTAAACCGTCGAGTTCGGCG	428	53.7/60	(Cattoir et al., 2007)
16S rRNA gene	GGTAGTCYAYGCMSTAAACG<br>GACARCCATGCASCACCTG	263	62/60	(Bach et al., 2002)

Table S2 Antibiotic concentration in different groups. ($\mu g\ kg^{-1}$ or $\mu g\ L^{-1}$)

Antibiotics	Control group		Treatment groups	
			tetracyclines group	
tetracyclines	Sediment	Water	Sediment	Water
chlorotetracycline	$3.5\times10^{1}\pm1.3\times10^{1}$	$1.2\times10^{-1}\pm6.5\times10^{-3}$	$3.1\times10^{3}\pm3.5\times10^{1}$	$3.0\times10^{-1}\pm9.7\times10^{-3}$
oxytetracycline	$2.0\times10^{1}\pm2.5\times10^{0}$	$3.6\times10^{-2}\pm6.2\times10^{-3}$	$8.8\times10^{3}\pm3.1\times10^{2}$	$2.9\times10^{-1}\pm7.2\times10^{-2}$
doxycycline	$4.2\times10^{1}\pm6.1\times10^{0}$	$9.4\times10^{-1}\pm5.2\times10^{-2}$	$1.1\times10^{4}\pm8.8\times10^{1}$	$5.5\times10^{-1}\pm7.2\times10^{-2}$
			sulfonamides group	
sulfonamides	Sediment	Water	Sediment	Water
sulfamethoxydiazine	$1.5\times10^{1}\pm2.0\times10^{0}$	$4.8\times10^{-1}\pm2.1\times10^{-1}$	$1.2\times10^{2}\pm4.2\times10^{0}$	$2.2\times10^{2}\pm1.5\times10^{1}$
sulfamethazine	$1.8\times10^{1}\pm3.5\times10^{0}$	$2.7\times10^{-1}\pm9.8\times10^{-2}$	$1.6\times10^{2}\pm7.5\times10^{0}$	$7.1\times10^{2}\pm4.3\times10^{1}$
sulfamethoxazole	$8.3\times10^{0}\pm7.4\times10^{-1}$	$6.9\times10^{-1}\pm2.0\times10^{-1}$	$1.4\times10^{2}\pm7.8\times10^{0}$	$1.7\times10^{2}\pm1.1\times10^{1}$
			fluoroquinolones group	
fluoroquinolones	Sediment	Water	Sediment	Water
enrofloxacin	$2.1\times10^{1}\pm4.0\times10^{0}$	$5.8\times10^{-2}\pm4.5\times10^{-3}$	$6.4\times10^{3}\pm4.7\times10^{1}$	$2.2\times10^{0}\pm7.1\times10^{-2}$
ciprofloxacin	$2.4\times10^{1}\pm4.6\times10^{0}$	$9.2\times10^{-2}\pm2.0\times10^{-2}$	$6.0\times10^{3}\pm1.1\times10^{2}$	$2.0\times10^{0}\pm1.2\times10^{-1}$
norfloxacin	$1.3\times10^{1}\pm4.2\times10^{0}$	$2.2\times10^{-1}\pm2.7\times10^{-3}$	$4.3\times10^{3}\pm8.8\times10^{1}$	$1.3\times10^{0}\pm8.3\times10^{-2}$

Table S3 Relative abundance[a] of ARGs[b] in different groups.

ARGs	Control group		Treatment groups	
			tetracyclines group	
tet resistance genes	Sediment	Water	Sediment	Water
tetM	$1.3\times10^{-3}\pm1.2\times10^{-4}$	$2.1\times10^{-3}\pm6.4\times10^{-5}$	$4.9\times10^{-3}\pm4.1\times10^{-4}$	$9.7\times10^{-2}\pm7.3\times10^{-3}$
tetO	$1.2\times10^{-3}\pm4.0\times10^{-5}$	$8.3\times10^{-4}\pm2.0\times10^{-4}$	$3.7\times10^{-3}\pm1.4\times10^{-4}$	$5.6\times10^{-2}\pm6.0\times10^{-3}$
tetW	$3.2\times10^{-2}\pm6.9\times10^{-4}$	$1.4\times10^{-3}\pm1.4\times10^{-4}$	$1.3\times10^{-1}\pm6.7\times10^{-3}$	$4.0\times10^{-2}\pm9.5\times10^{-3}$
tetQ	$4.2\times10^{-3}\pm4.9\times10^{-5}$	$2.6\times10^{-3}\pm4.5\times10^{-4}$	$1.9\times10^{-2}\pm8.3\times10^{-4}$	$1.6\times10^{-1}\pm2.3\times10^{-2}$
tetX	$1.3\times10^{-1}\pm2.9\times10^{-2}$	$4.9\times10^{-4}\pm1.1\times10^{-4}$	$2.3\times10^{-1}\pm8.9\times10^{-3}$	$7.7\times10^{-3}\pm1.5\times10^{-3}$
tetS	$5.3\times10^{-5}\pm2.0\times10^{-5}$	nd[c]	$1.1\times10^{-4}\pm3.4\times10^{-6}$	nd
tetB/P	nd	nd	nd	nd
			sulfonamides group	
sul resistance genes	Sediment	Water	Sediment	Water
sul1	$1.1\times10^{-2}\pm3.9\times10^{-4}$	$8.3\times10^{-3}\pm2.2\times10^{-4}$	$5.0\times10^{-2}\pm1.8\times10^{-2}$	$7.5\times10^{-2}\pm4.0\times10^{-3}$
sul2	$1.9\times10^{-2}\pm1.2\times10^{-3}$	$1.5\times10^{-2}\pm1.1\times10^{-3}$	$1.1\times10^{-1}\pm3.0\times10^{-2}$	$7.9\times10^{-2}\pm4.9\times10^{-4}$
sul3	$2.5\times10^{-3}\pm1.3\times10^{-4}$	$5.8\times10^{-4}\pm7.6\times10^{-5}$	$1.8\times10^{-2}\pm6.5\times10^{-3}$	$4.6\times10^{-4}\pm3.9\times10^{-5}$
			fluoroquinolones group	
PMQR genes	Sediment	Water	Sediment	Water
oqxA	$3.0\times10^{-3}\pm2.8\times10^{-4}$	$3.2\times10^{-4}\pm2.5\times10^{-5}$	$1.2\times10^{-2}\pm4.8\times10^{-3}$	$1.2\times10^{-3}\pm2.8\times10^{-4}$
oqxB	$1.0\times10^{-2}\pm5.3\times10^{-4}$	$9.2\times10^{-4}\pm1.2\times10^{-4}$	$4.2\times10^{-2}\pm3.4\times10^{-3}$	$1.6\times10^{-3}\pm3.6\times10^{-4}$
aac(6')-Ib	$2.6\times10^{-3}\pm1.8\times10^{-4}$	$2.2\times10^{-4}\pm4.4\times10^{-5}$	$7.0\times10^{-3}\pm1.7\times10^{-3}$	$1.7\times10^{-3}\pm4.0\times10^{-4}$
qnrS	$5.5\times10^{-4}\pm1.3\times10^{-4}$	nd	$1.0\times10^{-3}\pm2.3\times10^{-5}$	nd
qepA	nd	nd	nd	nd

[a] the copies of antibiotic resistance genes/the copies of 16S rRNA gene.

[b] ARGs: antibiotic resistance genes, PMQR genes: plasmid-mediated quinolone resistance genes.

[c] nd: not detected.

References

Aminov R., Garrigues-Jeanjean N., and Mackie R. (2001). Molecular ecology of tetracycline resistance: development and validation of primers for detection of tetracycline resistance genes encoding ribosomal protection proteins. *Appl. Environ. Microb.* 67, 22-32.

Bach H-J., Tomanova J., Schloter M., and Munch J. (2002). Enumeration of total bacteria and bacteria with genes for proteolytic activity in pure cultures and in environmental samples by quantitative PCR mediated amplification. *J. Microbiol. Meth.* 49, 235-245.

Cattoir V., Poirel L., Rotimi V., and Soussy C-J. (2007). Nordmann P. Multiplex PCR for detection of plasmid-mediated quinolone resistance *qnr* genes in ESBL-producing enterobacterial isolates. *J. Antimicrob. Chemoth.* 60, 394-397.

Kim H.B., Wang M., Park C.H., Kim E-C., Jacoby G.A., and Hooper D.C. (2009). *oqxAB* encoding a multidrug efflux pump in human clinical isolates of Enterobacteriaceae. *Antimicrob. Agents Ch.* 53, 3582-3584.

Ng L-K., Martin I., Alfa M., and Mulvey M. (2001). Multiplex PCR for the detection of tetracycline resistant genes. *Mol. Cell. Probe.* 15, 209-215.

Park C.H., Robicsek A., Jacoby G.A., Sahm D., and Hooper D.C. (2006). Prevalence in the United States of *aac (6')-Ib-cr* encoding a ciprofloxacin-modifying enzyme. *Antimicrob. Agents Ch.* 50, 3953-3955.

Pei R., Kim S-C., Carlson K.H., and Pruden A. (2006). Effect of river landscape on the sediment concentrations of antibiotics and corresponding antibiotic resistance genes (ARGs). *Water Res.* 40, 2427-2435.

Xia L-N., Li L., Wu C-M., Liu Y-Q., Tao X-Q., and Dai L., et al. (2010). A survey of plasmid-mediated fluoroquinolone resistance genes from *Escherichia coli* isolates and their dissemination in Shandong, China. Foodborne Pathog. Dis. 7, 207-215.

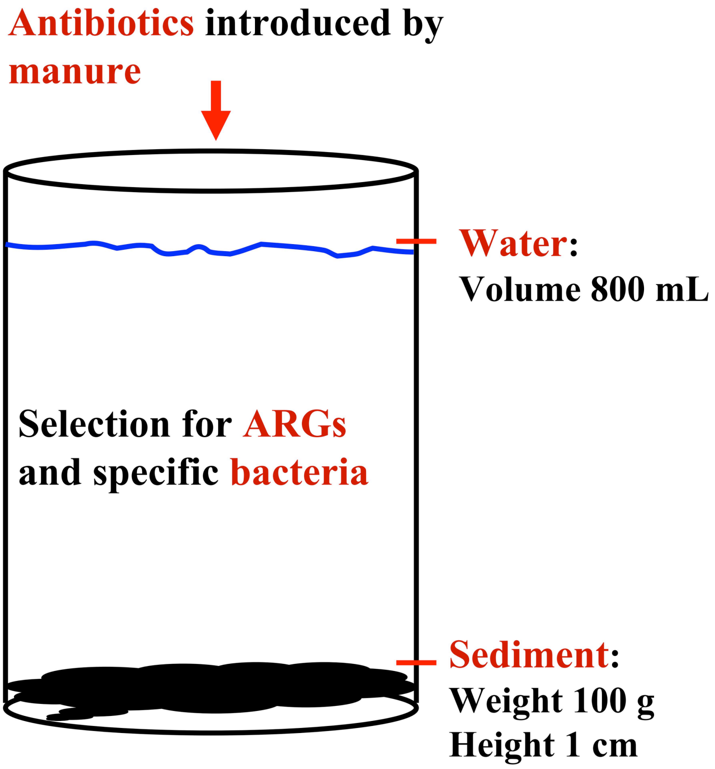

Fig. S1. Freshwater-sediment microcosms setup.
